# CXCR1 and CXCR2 are potential neutrophil extracellular trap-related treatment targets in ulcerative colitis: insights from Mendelian randomization, colocalization and transcriptomic analysis

**DOI:** 10.3389/fimmu.2024.1425363

**Published:** 2024-09-12

**Authors:** Yichuan Xv, Yiyi Feng, Jiang Lin

**Affiliations:** ^1^ Department of Gastroenterology, Longhua Hospital, Shanghai University of Traditional Chinese Medicine, Shanghai, China; ^2^ Department of Rheumatology, Yueyang Hospital of Integrated Traditional Chinese and Western Medicine, Shanghai University of Traditional Chinese Medicine, Shanghai, China

**Keywords:** Mendelian randomization, neutrophil extracellular trap, causal effect, ulcerative colitis, innate immunity

## Abstract

**Objectives:**

There is already substantial evidence indicating that neutrophil extracellular trap (NET) formation contributes to the inflammatory cascade in ulcerative colitis (UC). However, the precise regulatory mechanisms governing this process remain elusive. This study aimed to determine the role of NET-related genes in UC and reveal possible mechanisms.

**Methods:**

Employing a two-sample MR methodology, we investigated the correlations between NET-associated genes (NRGs) and UC with summary data derived from a genome-wide association study (12,366 cases vs. 33,609 controls) and FinnGen (8,279 cases vs. 261,098 controls). The main analysis employed the inverse variance weighted method, supplemented by the MR-Egger method and weighted median method. Sensitivity analysis was conducted to rule out the interference of heterogeneity and pleiotropy among utilized instrument variables. The colocalization analysis was used to determine whether the identified NRGs and UC shared casual variants. Cross-tissue expression analysis was performed to characterize the expression patterns of target NRGs, while multi-gene correlation analysis and GSEA analysis were conducted to explore the mechanisms by which target NRGs promote UC and NET formation. Immunohistochemistry was used to validate the protein expression of target NRGs in the colon tissue of UC patients.

**Results:**

After the validation of two datasets, seven NRGs were associated with the risk of UC. The higher expression of ITGB2 was associated with increased UC risk, while the expression of CXCR1, CXCR2, IRAK4, MAPK3, SIGLEC14, and SLC22A4 were inversely associated with UC risk. Colocalization analysis supported the correlation between CXCR1/2 and UC risk. Expression analysis indicated that CXCR1/2 were down-regulated in peripheral blood, but up-regulated in colon tissue. GSEA analysis and correlation analysis indicated that CXCR1/2 promoted UC and NET formation through neutrophil chemotaxis and PAD4-mediated pathways, separately. Immunohistochemical results confirmed the high expression of CXCR1/2 in colon tissues of UC patients.

**Conclusions:**

Our study identified CXCR1/2 as candidate targets in UC among all NRGs through multi-method argumentation, providing new insights of the regulation mechanisms of NET formation in the pathogenesis of UC.

## 1 Introduction

Ulcerative colitis (UC) is considered an inflammatory condition that targets the intestinal tract, initiating in the rectum and potentially extending through the entire colon. Clinically, UC manifests as persistent or recurring diarrhea, bleeding and abdominal pain, attributable to the inflammatory pathology (1).

Neutrophils are widely involved in the inflammatory response in UC (2). A notable characteristic of tissue damage in UC is the significant infiltration of neutrophils into the intestinal mucosa, which correlates with both endoscopic severity and systemic inflammatory indexes (3). Additionally, as for patients treated with biologics, the persistent infiltration of neutrophils in colon tissue at week 14 is associated with failure of endoscopic and histological healing at week 52 (4). The formation of neutrophil extracellular traps (NETs) is an important mechanism of neutrophil-induced inflammation, which effectively traps and eliminates invading pathogens in innate immune response (5). On the other hand, the constituents of NETs exhibit non-selective cytotoxicity and pro-inflammatory properties, potentially contributing to the immune dysregulation evident in UC (6). The peripheral blood serves as a monitoring mechanism for detecting tissue damage, providing insights into pathological occurrences across the entire human body, which is also the main environment for targeted drugs (7). A pilot study demonstrated higher levels of peripheral NETs in patients with UC compared to healthy individuals (8). However, the regulation mechanisms of NET formation involved in UC are still not fully understood. One study reported changes in the expression of NET-related genes (NRGs) in peripheral blood (9), but it could not be determined whether the dysregulation plays pathological roles or reflects the secondary effects of the illness or treatment. Identifying their pathogenic effects is crucial for understanding UC’s pathophysiology and developing new drug targets.

Mendelian randomization (MR) employed single nucleotide polymorphisms (SNPs) as instrumental variables (IVs) for the exposure (e.g., gene expression in peripheral blood) to conduct causal inference. The random allocation at conception and relative conservation of genetic variations over the course of diseases lend MR a reduced susceptibility to confounding influences (10). This feature helps overcome biases from environment and intrinsic limitation of observational studies. In this study, we used MR and colocalization analysis to explore the pathogenicity of NRG expression in peripheral blood on UC. Overall, these findings hold promise for offering valuable insights into elucidating the underlying mechanisms involved in the innate immunity of UC and offer potential avenues for its treatment.

## 2 Method

### 2.1 Study design

**Figure 1:**
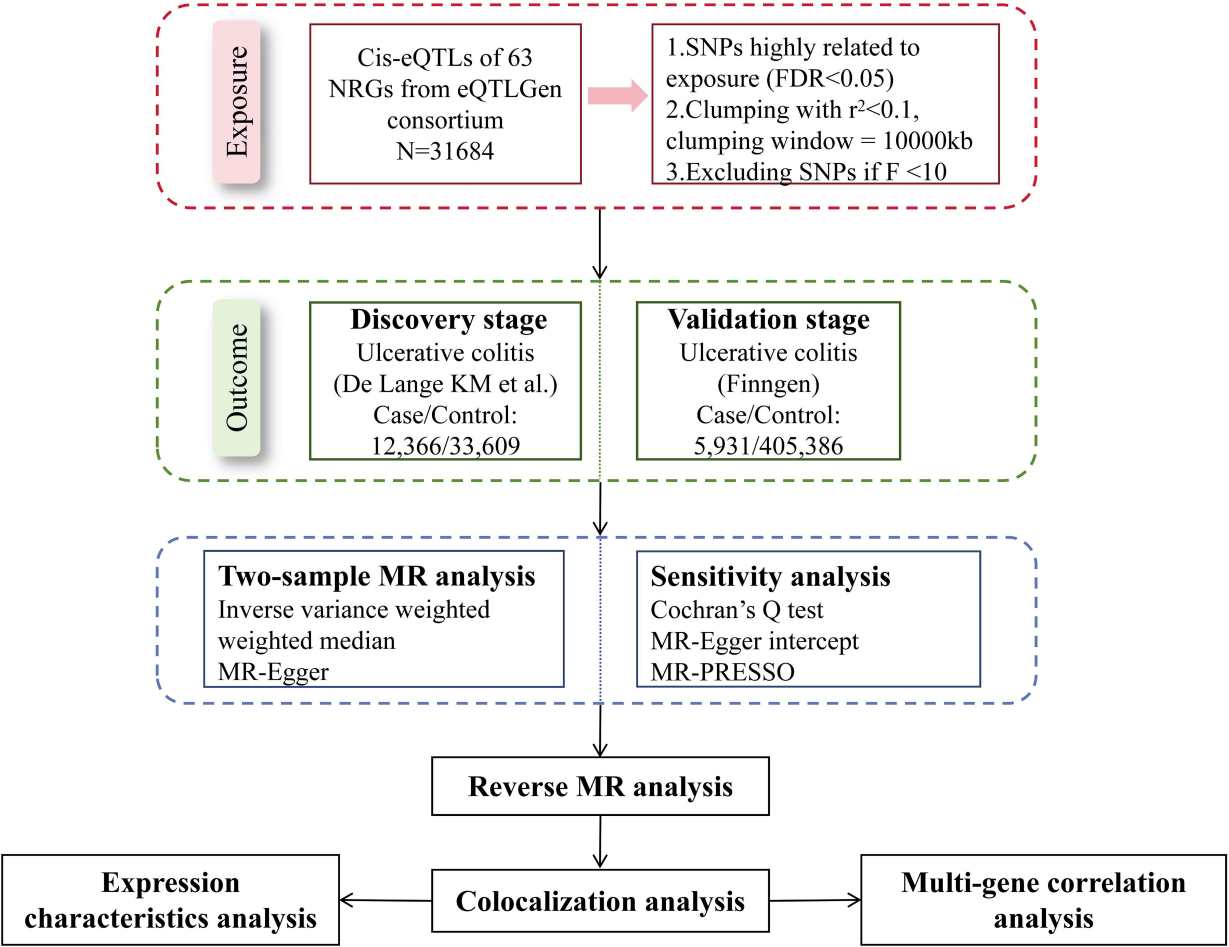
Schematic illustration of this MR study design.

We explored the causal relationships between NRGs and UC with the largest summary data available today. **Figure 1** presented the schematic illustration of study design. In our MR study, the genetic IVs were subject to three MR assumptions to obtain credible findings: (i) IVs must exhibit a strong association with exposure; (ii) IVs demonstrated no correlation with confoundings; (iii) IVs affected the outcome only through exposure (11). Only IVs that meet the first assumption can effectively represent exposure, which is a prerequisite for the establishment of MR studies. The establishment of the second and third assumptions ensures the purity of the causal relationship, that is, IVs only affect the outcome through exposure, excluding the influence of confounding factors and reverse causal associations on the conclusion. Our working code has been uploaded in a GitHub repository. Here is the link: https://github.com/wjjfromSCN/MendelianRandomizationAndBioinformaticAnalysis.

### 2.2 Exposure data

The available (eQTLs) of NRGs were mined from eQTLGen Consortium (https://eqtlgen.org/), which is the only open project providing eQTLs from European ancestry. In brief, the summary dataset from the eQTLGen consortium includes cis-eQTLs for 16,987 genes, derived predominantly from 31,684 blood samples of European ancestry. A comprehensive depiction of the data is available within the original literature (12). The list of NRG was from a previous study (13) (**Supplementary Table S1**). Exclusively cis-eQTLs were employed in this study, focusing within a 5kb proximity upstream or downstream of gene start and end points due to their direct influence on gene expression regulation. Among 69 genes, cis-eQTLs for 63 NRGs were finally obtained.

### 2.3 Outcome data

In the selection of outcome GWAS data, the sample size, inclusion criteria, and sequencing depth of the study cohort are our most reference indicators. The summary data of the UC discovery cohort came from the latest genome-wide association study (GWAS) because it included the largest number of patients. The study included 45,975 participants of mainly European ancestry (cases/controls: 12,366/33,609) (14). In this study, all UC patients strictly met the clinical criteria for UC, as determined by standardized evaluations including symptomatology, radiology, and endoscopy. The summary data of the replication cohort came from FinnGen Release 10 (https://www.finngen.fi/en), which included 5,931 cases and 405,386 controls (15). FinnGen’s GWAS data has been continuously updated, has a satisfactory sequencing depth, and provides a large number of available SNPs. All UC patients in the Finngen cohort met the ICD-10 diagnostic criteria.

### 2.4 SNP selection

Quality control was performed in this study to screen eligible SNPs. To minimize potential bias from population stratification, we ensured that the participants of exposure and outcome cohorts consisted predominantly of individuals of European descent. SNPs significantly associated with gene expression were selected first at the threshold of FDR < 0.05 (16, 17) and then were clumped with a linkage disequilibrium (LD) coefficient threshold of r^2^ = 0.1 and a window of 10,000kb to eliminate the interference of linkage disequilibrium on the results, according to a reference panel from the 1,000 Genome Project (18). Notably, we used a relatively loose threshold in SNP clumping because too strict clumping threshold would lose a large number of SNPs, leading to a lower statistic power of effect size. At the genomic level, it can also be considered that there is no linkage disequilibrium between IVs with the LD threshold of r^2 =^ 0.1, so it was acceptable to apply this threshold in subsequent MR analysis. Calculation of F-statistics were conducted for each SNP to exclude weak instrumental variables, and SNPs with F > 10 were considered valid IVs. The default parameters were applied in the reverse MR analysis. Firstly, SNPs related to UC were selected at the threshold of P<5e-8 and were clumped with r^2^ = 0.001 and a window of 10,000kb. Also, 1,000 Genome Project was used as the reference panel in the reverse MR analysis. Details of all SNPs used in our study were exhibited in **Supplementary Table S2**.

### 2.5 Statistical analysis

Firstly, we aligned the effect alleles of exposure and outcome before MR analysis. In this study, we mainly referred to the results of inverse variance weighted (IVW) method. In this method, the Wald ratio is calculated for each SNP to determine its effect on the outcome, and then are fitted using the inverse of the outcome variance (the square of the standard error) as the weight. In our study, the estimates of IVW method were calculated according to the following formula:


$${\hat{\beta }}_{ivw}=\frac{\sum \frac{{\hat{\beta }}_{exposure}{\hat{\beta }}_{outcome}}{var\left({\hat{\beta }}_{outcome}\right)}}{\sum \frac{{\hat{\beta }}_{exposure}^{2}}{var\left({\hat{\beta }}_{outcome}\right)}}$$

IVW assumes the validity of all genetic instruments and the directly proportional relationship between outcome and exposure (10). Because the intercept term is not considered in the calculation process of the IVW method, the included SNPs must be ensured not to have pleiotropy, otherwise the results would be greatly biased. Accordingly, we subsequently performed sufficient sensitivity analyses to ensure the robustness of the IVW results. Additional methods were utilized to complement the IVW results. The weighted median (WM) method offers robust outcomes when valid instrumental variables are lacking, even if half of the data may originate from invalid instruments (19). The MR-Egger method, applying the linear regression algorithm, estimates an intercept to test horizontal pleiotropy, albeit with lower statistical efficiency (20).

We carried out additional sensitivity tests to assess the reliability of results. Cochran’s Q test was used to assess heterogeneity. Pleiotropy, consisting of vertical pleiotropy and horizontal pleiotropy, is the most important factor that determines whether the results of MR analysis are valid. The vertical pleiotropy reflects the downstream effects of exposure and does not bias MR results, that is unnecessary to be focused on in MR analysis. Horizontal pleiotropy was assessed using MR-Egger intercept test and Mendelian randomization pleiotropy residual sum and outlier (MR-PRESSO). In addition, MR-PRESSO provides an outlier test to determine the robustness of the study results. Leave-one-out analysis was additionally employed to validate the robustness of MR results. In Leave-one-out analysis, MR analysis was repeated after excluding SNPs one by one to determine whether the results are significantly disturbed by single SNPs (20, 21).

In the discovery cohort, to control type I error, only results whose *P*-value survived Benjamini and Hochberg (B-H) correction would be considered significant. The causality of all NRGs was also validated repeatedly in the FinnGen cohort, and the same B-H correction was used to filter significant NRGs. All MR analyses were conducted using the Two-Sample MR (version 0.5.6) package in R.

### 2.6 Colocalization analysis

To ascertain the existence of shared causal factors between significant NRGs and UC within a given chromosomal region, Bayesian colocalization analysis was performed using the R package coloc (22). The Bayesian method assessed the support corresponding to one of five exclusive hypotheses: H0, no association with either trait; H1, association with NRG expression only; H2, association with UC risk only; H3, association with both traits, but causal variants are different for two traits; H4, association with both traits and the causal variant is shared by both traits. The default prior probability was set at 1×10^−5^, which mean that SNPs within the given genomic region were significantly associated with NRG expression and UC risk. To obtain higher statistical power, we used GWAS summary data from the discovery cohort for colocalization analysis because of its larger amount of included UC patients. SNPs located within 500kb upstream and downstream of NRGs were used to performed colocalization analysis. The significance threshold for colocalization was designated at posterior probability (PP) of H4 > 0.80, and NRGs that colocalized with UC could be regarded as candidate targets for UC.

### 2.7 The expression characteristics of target NRGs in UC

Using the GTEx Portal, we initially analyzed expression levels of target NRGs in whole blood and gastrointestinal tract of healthy people. To gather expression characteristics of target NRGs in peripheral blood and colon of UC patients, bulk RNA-sequencing data of peripheral blood (GSE169568) and colon tissues (GSE66407) from Gene Expression Omnibus (GEO) database were utilized, separately. In dataset GSE169568, RNA microarray data was acquired from 58 peripheral blood samples from treatment-näive UC patients and 95 healthy controls. In dataset GSE66407, RNA-sequencing data were from 62 gut biopsies of UC patients and 99 gut biopsies of controls. The expression counts from the original gene matrix were further normalized to obtain log_2_ fold change (FC) of each gene. B-H correction mentioned above was samely used to correct the *P*-value of each gene. Genes with adjusted *P*-value < 0.05 and log_2_ FC > 1 were considered as significant differentially expressed genes (DEGs).

### 2.8 Multi-gene correlation analysis

To further investigate the mechanism underlying the induction of NETs formation by identified NRGs in the colon of UC patients, we conducted a multi-gene correlation analysis to assess the co-expression relationships between significant NRGs and genes encoding NET marker proteins (ELANE, PADI4, MPO) in UC patients based on the GSE66407 dataset.

### 2.9 Gene set enrichment analysis

Based on the GSE66407 dataset, we further divided patients into high and low expression groups according to the expression of target NRGs, and gene set enrichment analysis (GSEA) was used to elucidate the coordinated alterations at the pathway level between the two groups. Applying GSEA analysis, we can further illustrate the mechanism by which the target NRGs mediate the inflammatory response and NET formation in UC. We used R package “org. Hs. Eg. Db” for acquiring Entrez ID of each DEG and “clusterProfiler” for enrichment analysis.

### 2.10 Immunohistochemistry

Colon tissue from UC patients was taken from patients undergoing colonoscopy and pathological examination at Longhua Hospital in Shanghai, China. The colon biopsies were fixed in 4% paraformaldehyde immediately after acquisition. The Ethics Review Committee of Shanghai Longhua Hospital in China approved the collection of human colon tissue and its use in our study (ethical approval number: 2023LCSY110). The guiding principles of the Declaration of Helsinki were followed in the conduct of the research. Written informed permission from each recruited patient was obtained. The primary antibodies of CXCR1 and CXCR2 were purchased from Nanjing Bioworld Company, China, and the general secondary antibodies and DAB solution were purchased from Fuzhou Maixin Company, China. The experimental process of immunohistochemistry staining was carried out according to the previous study (23). Paraffin pieces of the colon tissue were first baked at 60°C and then immersed in xylene, ethanol, and ultra-pure water for dewaxing. After blocking with 10% bovine serum albumin, the sections were incubated with primary antibody at 1:200 dilution overnight and then with secondary antibody for 1 hour. After treatment with DAB, the sections were observed under a 200× magnification microscope. Image J was used for quantitative analysis of the results.

## 3 Results

### 3.1 Preliminary MR analysis identified 13 NRGs associated with UC risk

**Figure 2:**
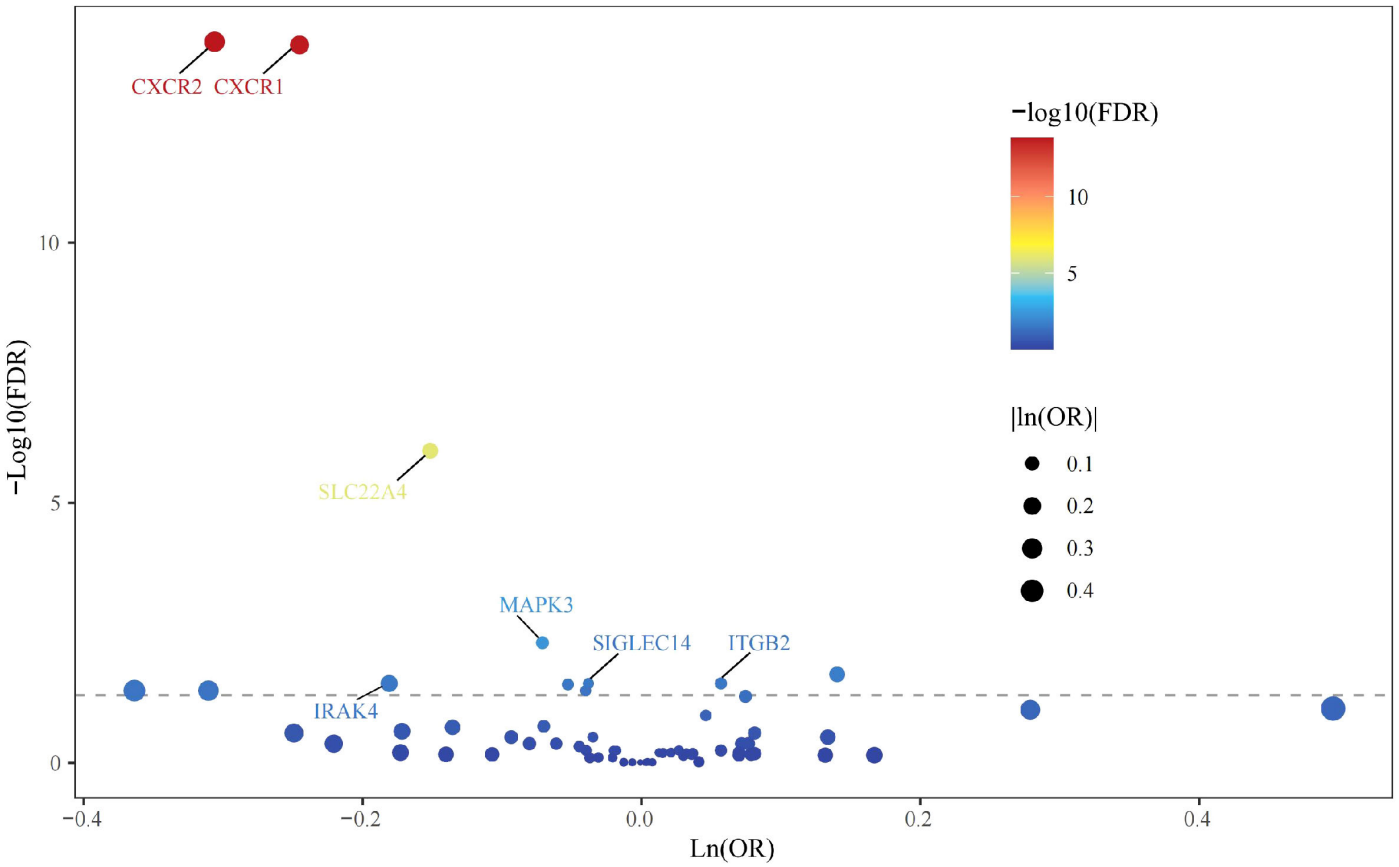
Volcano plot of MR results in the discovery stage.

**Figure 3:**
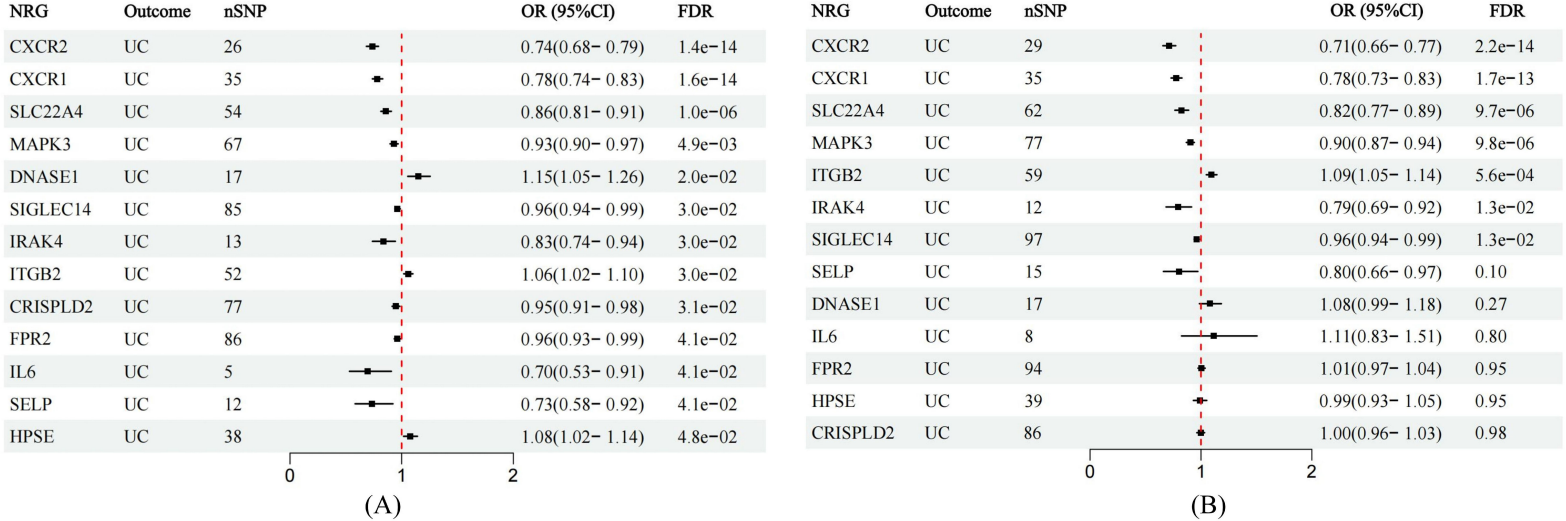
Forest plots showing the MR estimates of causal NRGs in the discovery and validation stage. **(A)** MR estimates for significant NRGs in discovery stage. **(B)** MR results in the validation set for significant NRGs identified in the exploration stage.

All cis-eQTLs of 63 NRGs utilized in this MR study were listed in **Supplementary Table S2**. In the exploratory set, after applying B-H correction, IVW results suggested causal relationships between 13 NRGs and UC (**Figure 2**). The expression of three NRGs in peripheral blood positively correlated with the risk of UC, while the expression of 11 NRGs was negatively associated with UC risk (**Figure 3A**). All statistic methods generated similar estimates, suggesting the reliability of the results (**Supplementary Table S3**). The F-statistics for SNPs of all NRGs were over ten, indicating minimal weak instrument bias. Besides, no heterogeneity or pleiotropy would interfere the results according to sensitivity analysis (**Supplementary Table S4**). Leave-one-out analysis excluded significant interference by any single SNP (**Supplementary Figure S1**).

### 3.2 The causal effects of seven NRGs were copied in validation stage

In the validation phase, we repeatedly assessed the causal influence of all NRGs on UC risk using the summary data from Finngen. MR analysis was performed employing identical methodology as utilized in the discovery cohort. The causal relationships between seven genes and UC were replicated, namely CXCR1, CXCR2, IRAK4, ITGB2, MAPK3, SIGLEC14, and SLC22A4. The higher ITGB2 expression was associated with increased UC risk, while the expression of CXCR1, CXCR2, IRAK4, MAPK3, SIGLEC14, and SLC22A4 were inversely associated with UC risk (**Figure 3B**). Sensitivity analysis ruled out heterogeneity or pleiotropy among the SNPs of these genes (**Supplementary Table S5**). Leave-one-out analysis confirmed the robustness of the results (**Supplementary Figure S2**). The direction of causal estimates derived from the discovery set and the verification set were consistent (**Figure 3**).

### 3.3 Reverse MR analysis ruled out inverse causal association

**Figure 4:**
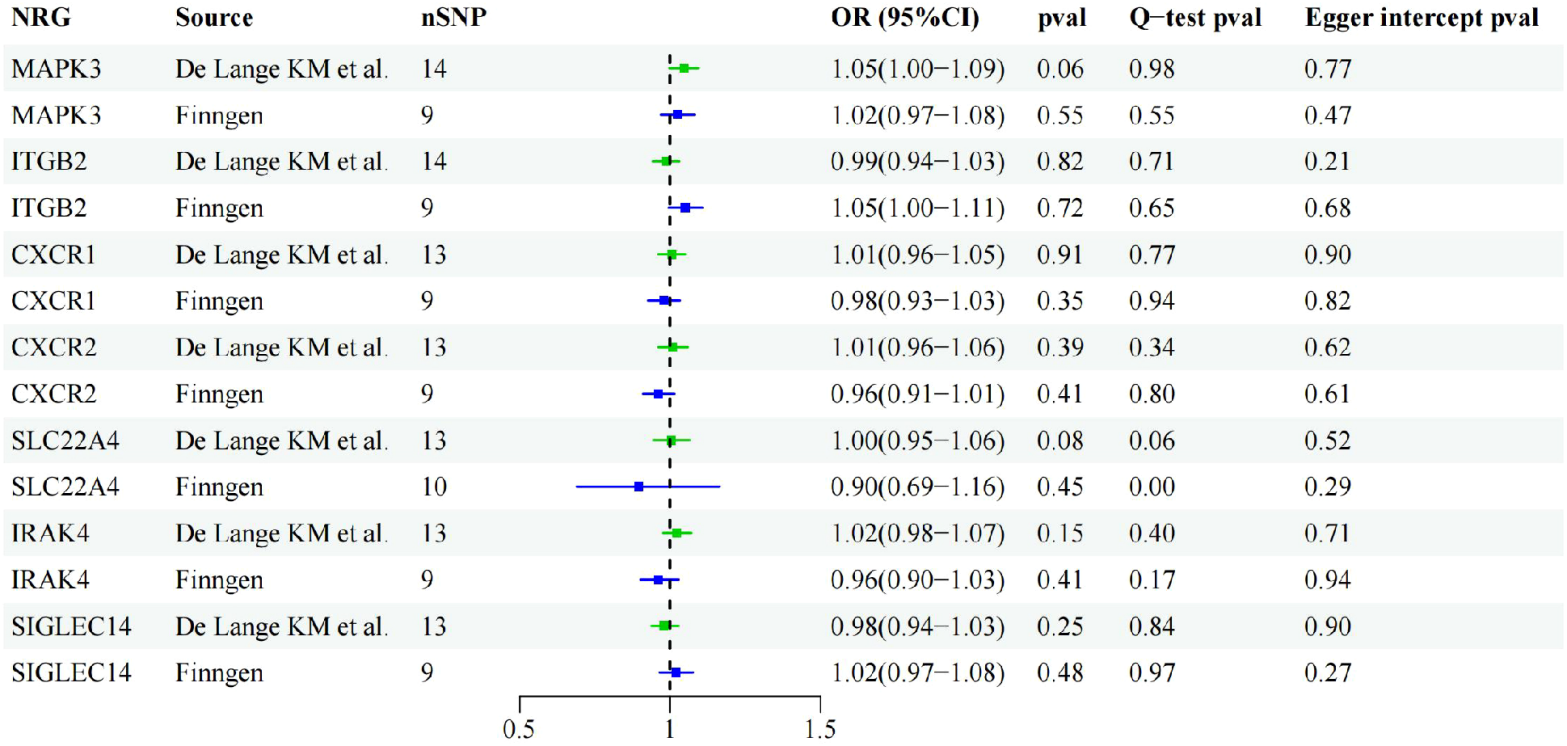
Forest plot of reverse MR results of identified NRGs.

Through cross-validation of the two datasets, we identified seven genes that may be associated with UC risk. To explore the potential reverse causality of each NRG with UC, we conducted reverse MR analysis on these seven NRGs using both discovery and validation datasets. The results showed that the *P-*values of all NRGs were above 0.05 in both GWAS from De Lange KM and the Finngen database, indicating the absence of any inverse causal relationship. Sensitivity analysis results further confirmed the absence of pleiotropy or heterogeneity in the reverse MR analysis (**Figure 4**).

### 3.4 Colocalization analysis supported the causal effects of CXCR1 and CXCR2

**Figure 5:**
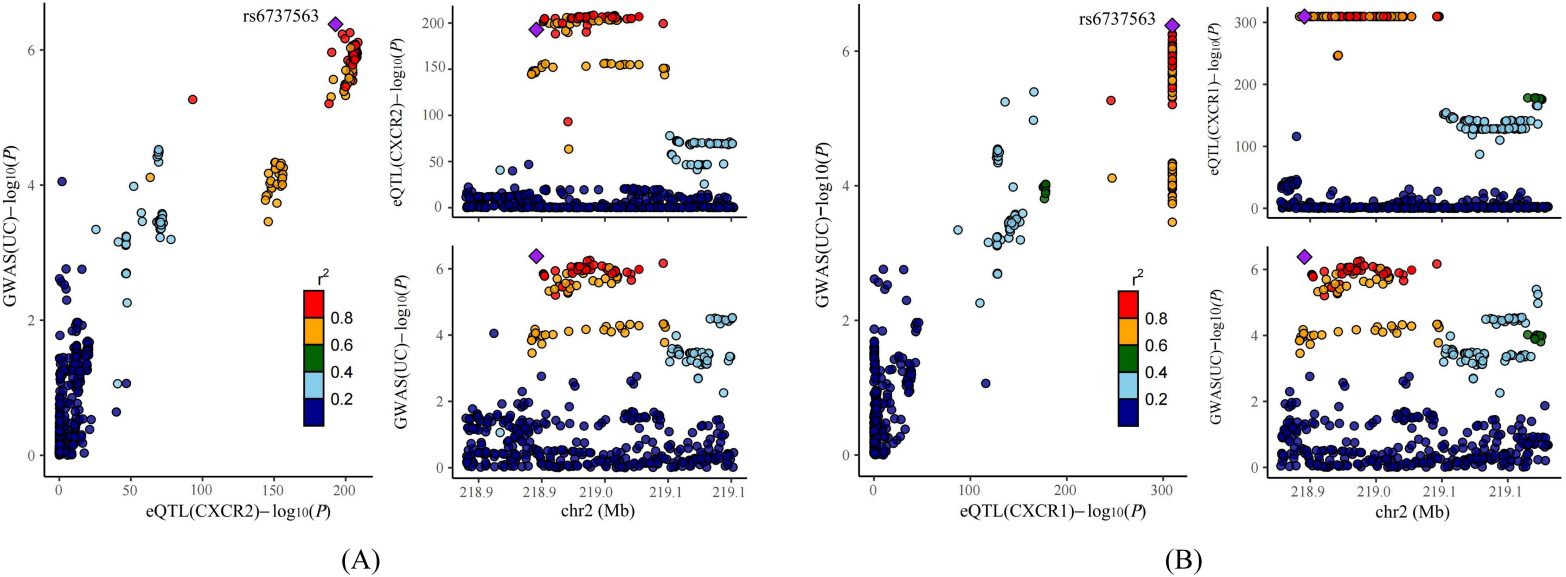
Results of colocalization analysis. **(A)** Colocalization analysis for eQTLs of CXCR2 and UC. **(B)** Colocalization analysis for eQTLs of CXCR1 and UC.

Previous studies have indicated that false positive MR results may emerge from a genetic locus where the associations between SNP-exposure and SNP-outcome stem from two distinct causal SNPs because of close linkage disequilibrium (24). Therefore, we carried out colocalization analysis of the seven causal genes obtained through the above analysis to investigate if exposure and outcome share the identical causal SNP. **Table 1** presented the results of the colocalization analysis. Among the seven NRGs examined, CXCR1 and CXCR2 exhibited strong evidence of colocalization with UC (PP.H4 > 0.8), indicating a shared causal SNP between the exposure and outcome. However, no evidence of colocalization was found for the other five NRGs. Furthermore, the colocalization analysis revealed that rs6737563 was the shared variant driving causal effects in both the CXCR1-UC and CXCR2-UC associations, as illustrated in **Figure 5**.

**Table 1:** Results of colocalization for 7 NRGs.

	CXCR1	CXCR2	IRAK4	ITGB2	MAPK3	SIGLEC14	SLC22A4
PP.H0	<0.001	<0.001	<0.001	<0.001	<0.001	<0.001	<0.001
PP.H1	0.002	<0.001	0.762	0.868	0.719	0.911	0.083
PP.H2	<0.001	<0.001	<0.001	<0.001	<0.001	<0.001	<0.001
PP.H3	0.145	0.043	0.037	0.088	0.134	0.075	0.892
PP.H4	0.852	0.956	0.201	0.043	0.147	0.014	0.025

### 3.5 Expression characteristics of CXCR1 and CXCR2

**Figure 6:**
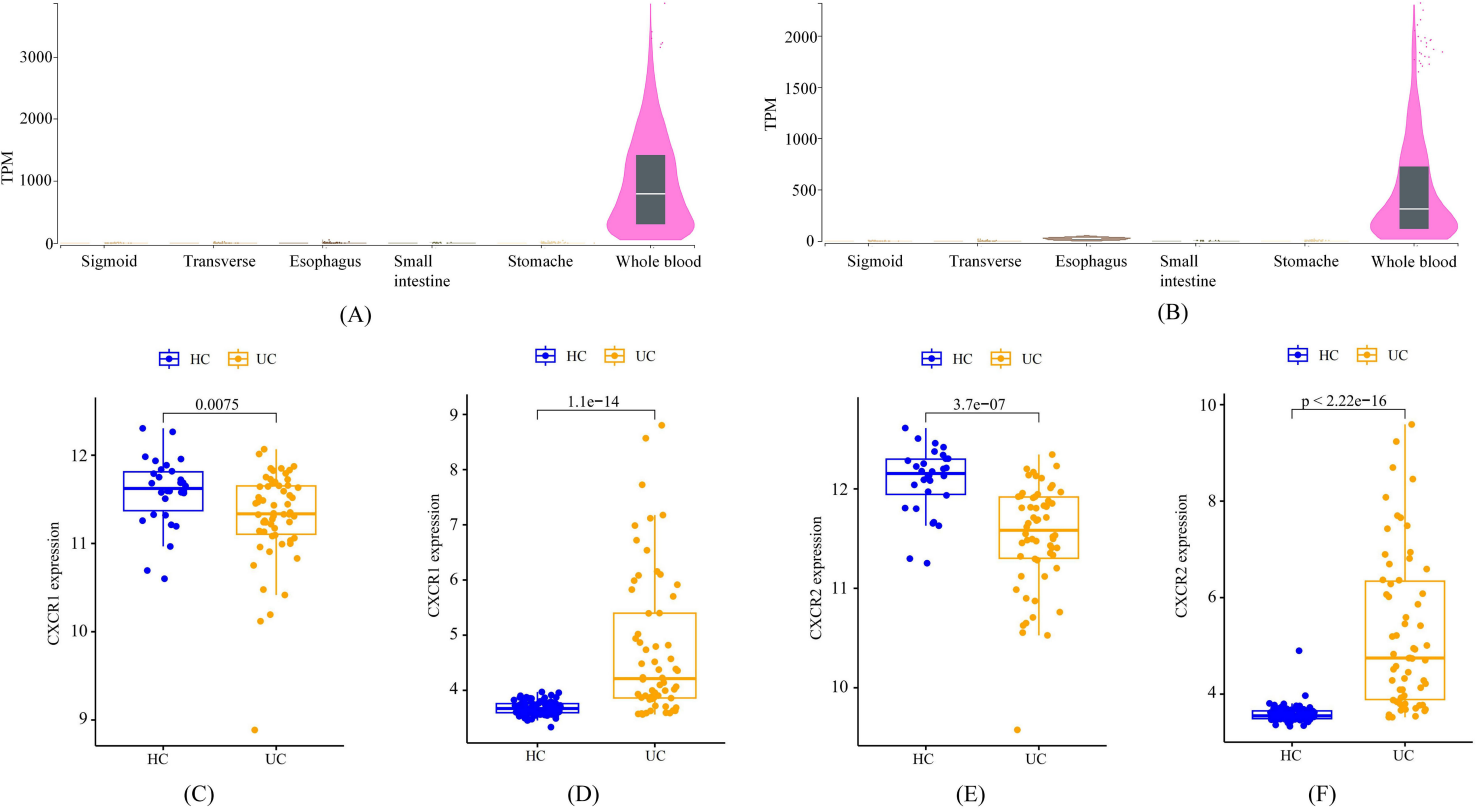
The expression characteristics of CXCR1 and CXCR2 under various conditions. **(A)** cross-tissue expression of CXCR1 in healthy individuals **(B)** cross-tissue expression of CXCR2 in healthy individuals **(C)** peripheral expression of CXCR1 in UC patients **(D)** colon expression of CXCR1 in UC patients **(E)** peripheral expression of CXCR2 in UC patients **(F)** colon expression of CXCR2 in UC patients.

According to data from the GTEx Portal, CXCR1 and CXCR2 exhibited predominant expression in peripheral blood, with minimal expression in the gastrointestinal tract (**Figures 6A, B**). Analysis of transcriptome data from GEO confirmed a significant decrease in the expression of CXCR1 and CXCR2 in peripheral blood among UC patients, in accordance with the outcomes of the MR analysis (**Figures 6C, E**). Interestingly, contrasting results were found in the colon mucosa of UC patients, where the expression of CXCR1 and CXCR2 significantly increased compared to healthy controls, indicating an opposing trend to that observed in peripheral blood (**Figures 6D, F**).

### 3.6 The mechanism by which CXCR1/2 promote UC onset and NET formation

**Figure 7:**
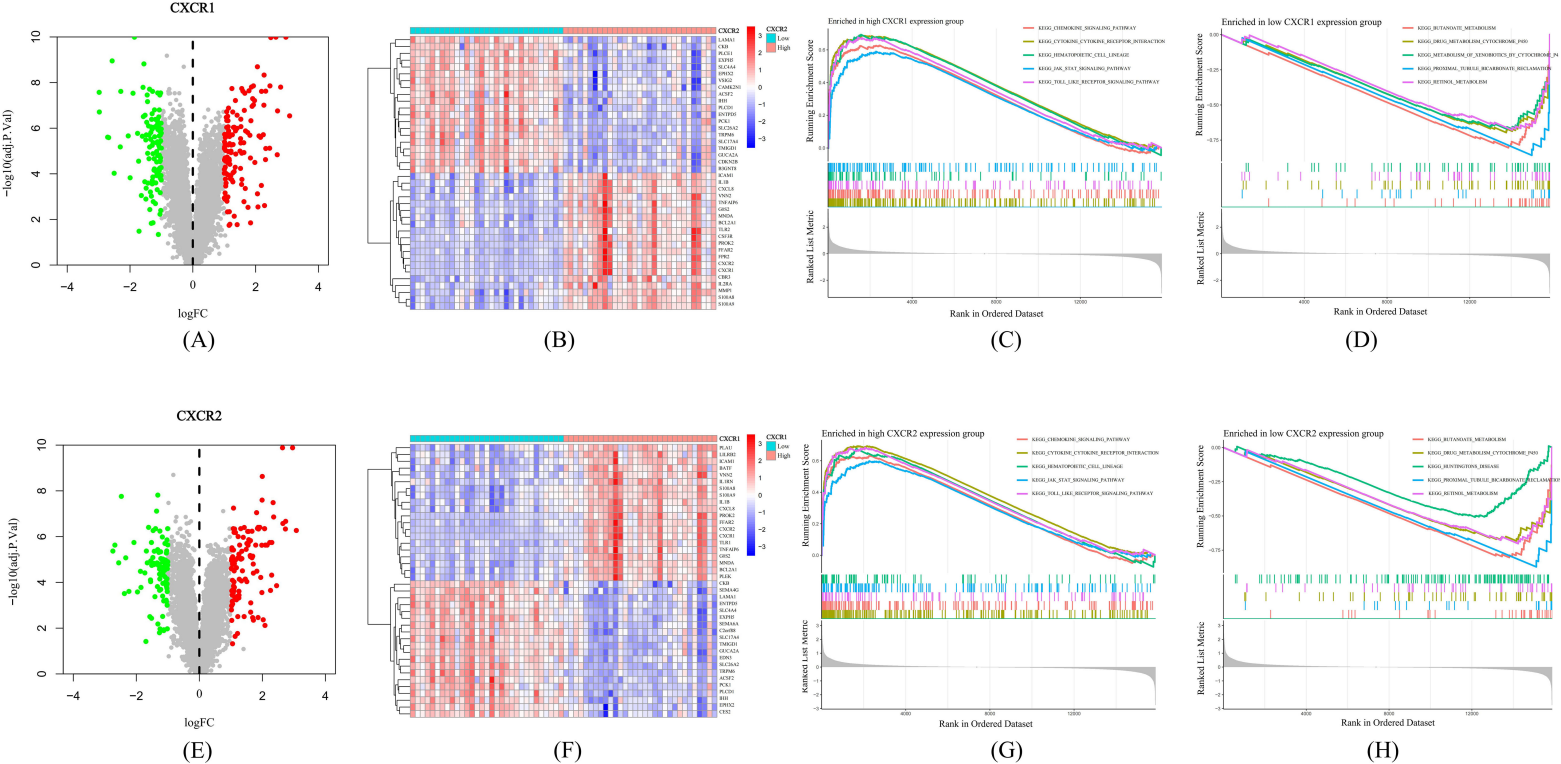
Mechanisms by which CXCR1/2 promote UC. **(A)** Differentially expressed genes between high expression group and low expression group according to CXCR1 expression. **(B)** Heatmap of the top 40 differentially expressed genes between two groups divided into according to CXCR1 expression. **(C)** The top five pathways enriched in high CXCR1 expression group. **(D)** The top five pathways enriched in low CXCR1 expression group. **(E)** Differentially expressed genes between high expression group and low expression group according to CXCR2 expression. **(F)** Heatmap of the top 40 differentially expressed genes between two groups divided into according to CXCR1 expression. **(G)** The top five pathways enriched in high CXCR2 expression group. **(H)** The top five pathways enriched in low CXCR2 expression group.

Based on GEO dataset GSE66407, we divided UC patients into two groups according to the expression of CXCR1/2. We identified a large number of DEGs between the high-expression and low-expression groups (**Figures 7A, E**), indicating that CXCR1/2 were uniquely involved in the pathogenesis of UC. **Figures 7B, F** exhibited the top 40 DEGs. We performed GSEA analysis to further explore synergistic changes in pathways between the two groups. The results of enrichment analysis were showed in **Figures 7C, D, G, H**. According to GSEA analysis, CXCR1/2 not only mediated strong neutrophil chemotaxis through ligand-receptor interactions, but also had a close relationship with the activation of inflammatory signaling such as JAK-STAT pathway and Toll-like receptor pathway.

**Figure 8:**
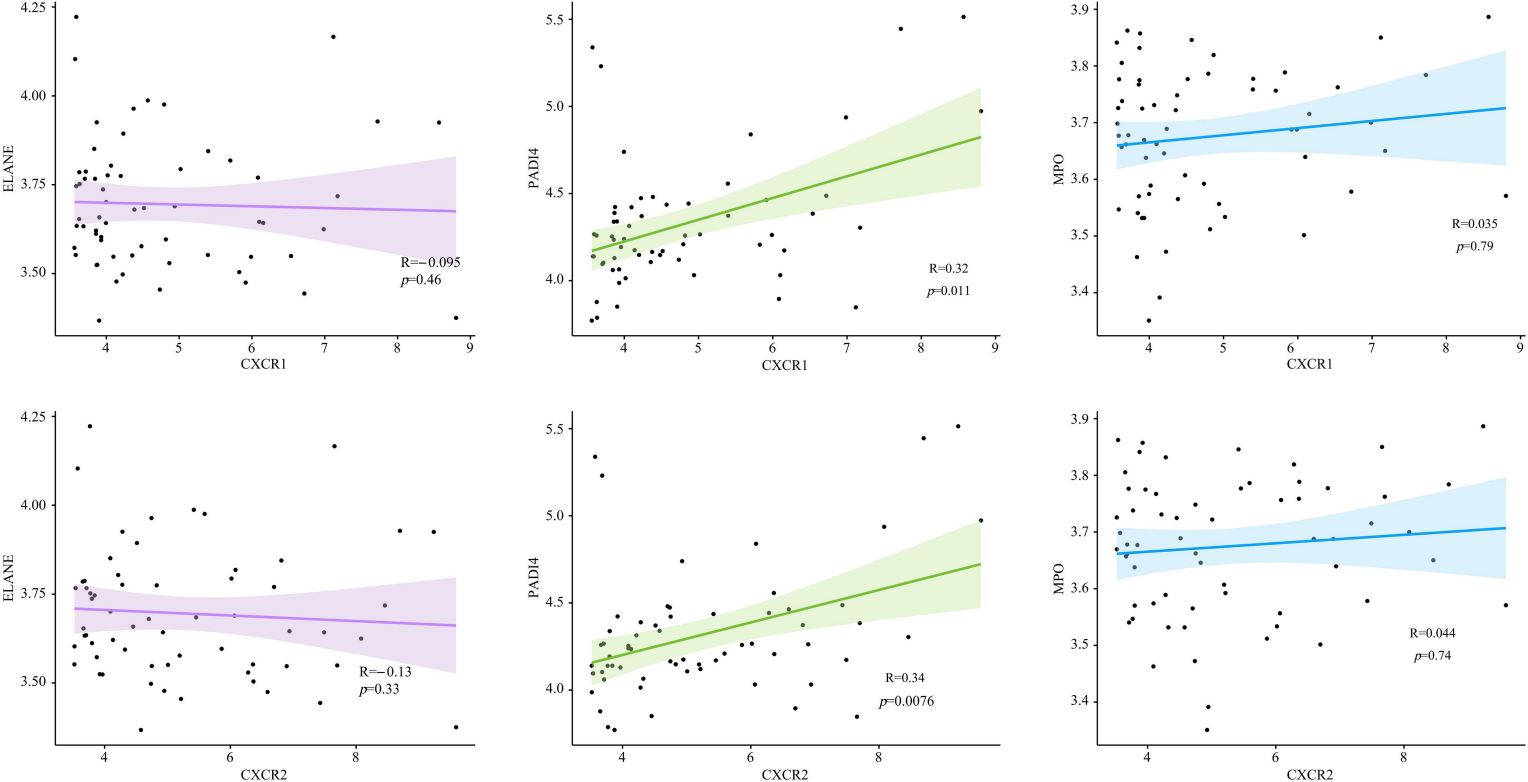
The expression correlation between CXCR1, CXCR2 and encoding genes of NET marker proteins in UC patients.

To find out how CXCR1/2 induce NET formation in the inflamed colon, we investigated the expression correlation between CXCR1 and CXCR2 with the encoding genes of NET marker proteins in colon tissues. Remarkably, we found a significant positive correlation between the expression of CXCR1 (R=0.34, *P*<0.05) and CXCR2 (R=0.32, *P*<0.05) with the expression of PADI4. However, no notable correlation was detected between the expressions of CXCR1 and CXCR2 and the genes of other NET marker proteins (MPO and ELANE) (**Figure 8**). These findings suggested that CXCR1 and CXCR2 may induce NET formation in UC by regulating the expression of PADI4.

### 3.7 Verification of the protein expression of CXCR1/2 in UC

**Figure 9:**
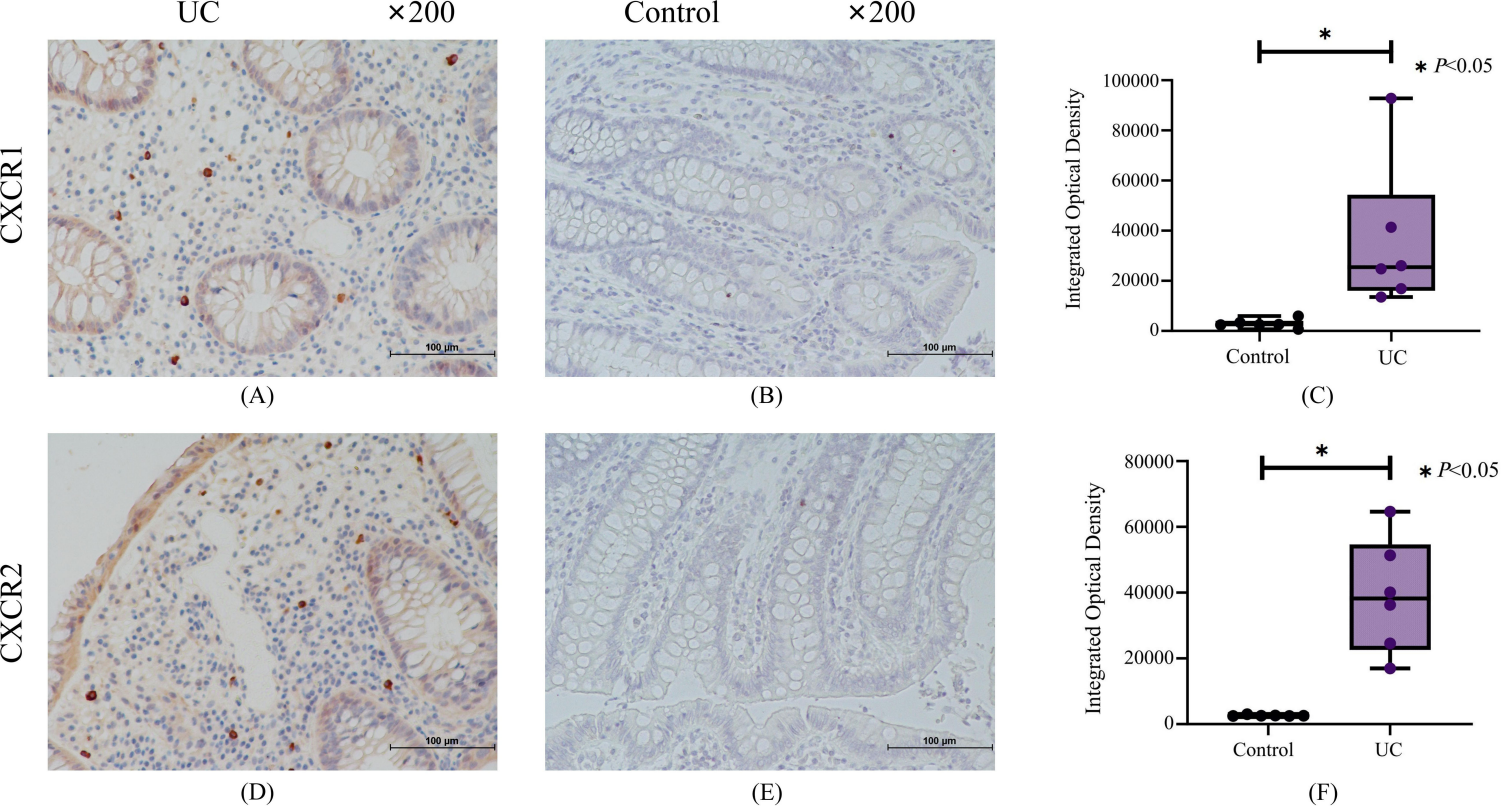
The results of immunohistochemistry for CXCR1 and CXCR2. **(A)** Representative immunohistochemistry staining for CXCR1 expressed in UC group. **(B)** Representative immunohistochemistry staining for CXCR1 expressed in control group. **(C)** The results of quantitative analysis for CXCR1. **(D)** Representative immunohistochemistry staining for CXCR2 expressed in UC group. **(E)** Representative immunohistochemistry staining for CXCR2 expressed in control group. **(F)** The results of quantitative analysis for CXCR2.

We applied immunohistochemistry with gut biopsies of UC patients to confirm our results. Immunohistochemical results indicated that compared with the normal control group, the crypt structure of UC patients was disorganized and the epithelium was destroyed, with a large number of inflammatory cells infiltrated in mucosal layer (**Figures 9A, B**). The expression of CXCR1/2 in colon tissue of UC patients was significantly higher than that in normal group (**Figure 9**).

## 4 Discussion

Neutrophil has been proved to be prominently involved in the promotion of UC through mechanisms like chemotaxis, the production of reactive oxygen species, and the release of NET (25). NETs includes a structure of DNA embedded with histones and cytotoxic proteins derived from neutrophils, released upon neutrophil activation, potentially leading to tissue damage if uncontrolled (26). Elevated levels of NET-associated proteins, such as myeloperoxidase, MMP-9, and neutrophilic defensins, have been observed in intestinal biopsies from UC patients, indicating their involvement in the disease pathogenesis (27). Moreover, NETs contribute to thrombotic events during active UC by inducing a procoagulant phenotype in platelets and endothelial cells (8). Although NET formation is strongly featured in UC, the corresponding regulatory mechanisms are relatively understudied, limiting the translational potential of targeting NET formation. Some pre-clinical results showed that peptidyl arginine deiminase 4 (PAD4) inhibitors and DNase I can relieve experimental colitis through inhibiting NET formation or increasing NET degradation (27, 28). However, their effectiveness was merely proved in small-scale pre-clinical studies, lacking support from evidence of larger scale studies (29).

Integrating eQTL, GWAS and transcriptome data, we revealed that CXCR1/2 is an important NET-related UC target on an epidemiological scale and revealed its possible mechanism of action. CXCR1 and CXCR2 are chemokine receptors predominantly expressed on neutrophils’ surface, facilitating neutrophil homing to inflammatory sites when activated by corresponding ligands like CXCL6 and CXCL8, pivotal in inflammation pathophysiology (30). Samaneh K et al. demonstrated the pathogenic role of CXCL8/CXCR1 axis in UC with an induced human UC-derived organoid (iHUCO) model (31). Bulk RNA-sequencing revealed transcriptional variations predominantly enriched in CXCL8/CXCR1 signaling. Dual IF staining for CXCL8/CXCR1 further confirmed their pathogenic roles. Moreover, over-expression of CXCL8/CXCR1 resulted in disrupted adherent junction patterns in epithelial cells, which may be the resultant impact of this inflammatory signaling in UC development. Another study found that the ligands of CXCR2 increased in inflamed colonic mucosa, and the deletion of CXCR2 diminished AOM/DSS-induced inflammation in colon. Intriguingly, while CXCR2 alteration did not affect dendritic cell, T cell, NK cell, and NKT cell infiltration, it showed a trend towards reducing neutrophil infiltration, particularly in acute colonic inflammation, indicating CXCR2’s primary involvement in neutrophil-mediated inflammation in UC (32). In our study, we found that CXCR1/2 were both associated with the activation of inflammatory pathways such as JAK/STAT pathway and Toll-like receptor pathway. The pathogenic role of JAK/STAT pathway in UC has been widely recognized, and the corresponding target therapy has been widely administrated in clinical practice (33). Although JAK inhibitors bring new treatment options to patients, they also increase the risk of thrombosis and tumors (33). In contrast, previous studies have shown that NET components can promote thrombotic tendencies in UC and colitis-related colon cancer. Inhibiting NET formation will significantly improve the above trends (8, 23). Therefore, blocking NET-related targets may be less susceptible to side effects such as thrombosis or tumors than JAK. Toll-like receptors (TLRs) play an important role in mediating innate immunity and adaptive immunity. Previous studies have shown that the components of neutrophil extracellular traps can directly stimulate the differentiation of Th17 cells through TLR2 pathway even at the absence of inflammatory microenvironment composed of inflammatory factors like IL-23, IL-1β and IL-6 (34). With the revolution in the treatment of UC, histological healing has become a new clinical goal for UC. Patients who achieve histological healing often have a better prognosis. Previous studies have shown that the degree of neutrophil infiltration in the colon, rather than lymphocytes, is an important histological indicator affecting the recurrence, surgical rate, and canceration rate of UC (4). Considering that CXCR1/2 plays an important role in the formation of NETs and the activation of immune response, targeting CXCR1/2 to improve histological inflammation is a promising therapeutic strategy.

Although current evidence underscores CXCR1 and CXCR2’s pivotal roles in promoting colonic inflammation, interestingly, we found that reduced levels of CXCR1 and CXCR2 in peripheral blood are correlated with the development of UC. It has previously been shown that peripheral CXCR1 and CXCR2 expression is consistently suppressed in response to inflammation signaling (35), which may result from the depletion of peripheral neutrophils expressing high CXCR1 or CXCR2 due to neutrophils’ migration to the site of inflammation. It has been previously reported that the expression of CXCL8, the ligand of CXCR1 and CXCR2, is increased in colonic tissues of UC patients, along with our findings of increased expression of CXCR1/2 in colon tissue, suggesting the existence of the neutrophil migration induced by CXCL8 signaling pathway. Previous findings that CXCR2 depletion strongly affects neutrophil infiltration of the colonic mucosa in mice with experimental colitis strengthens the evidence of this explanation. In addition, downregulation of the CXCR1/2 can also be caused by the action from miRNAs in activated neutrophils. miR-335-5-p is up-regulated in peripheral blood of UC patients (36), and has been previously evidenced to interact with CXCR2 mRNAs, leading to inhibition of its expression (37).

**Figure 10:**
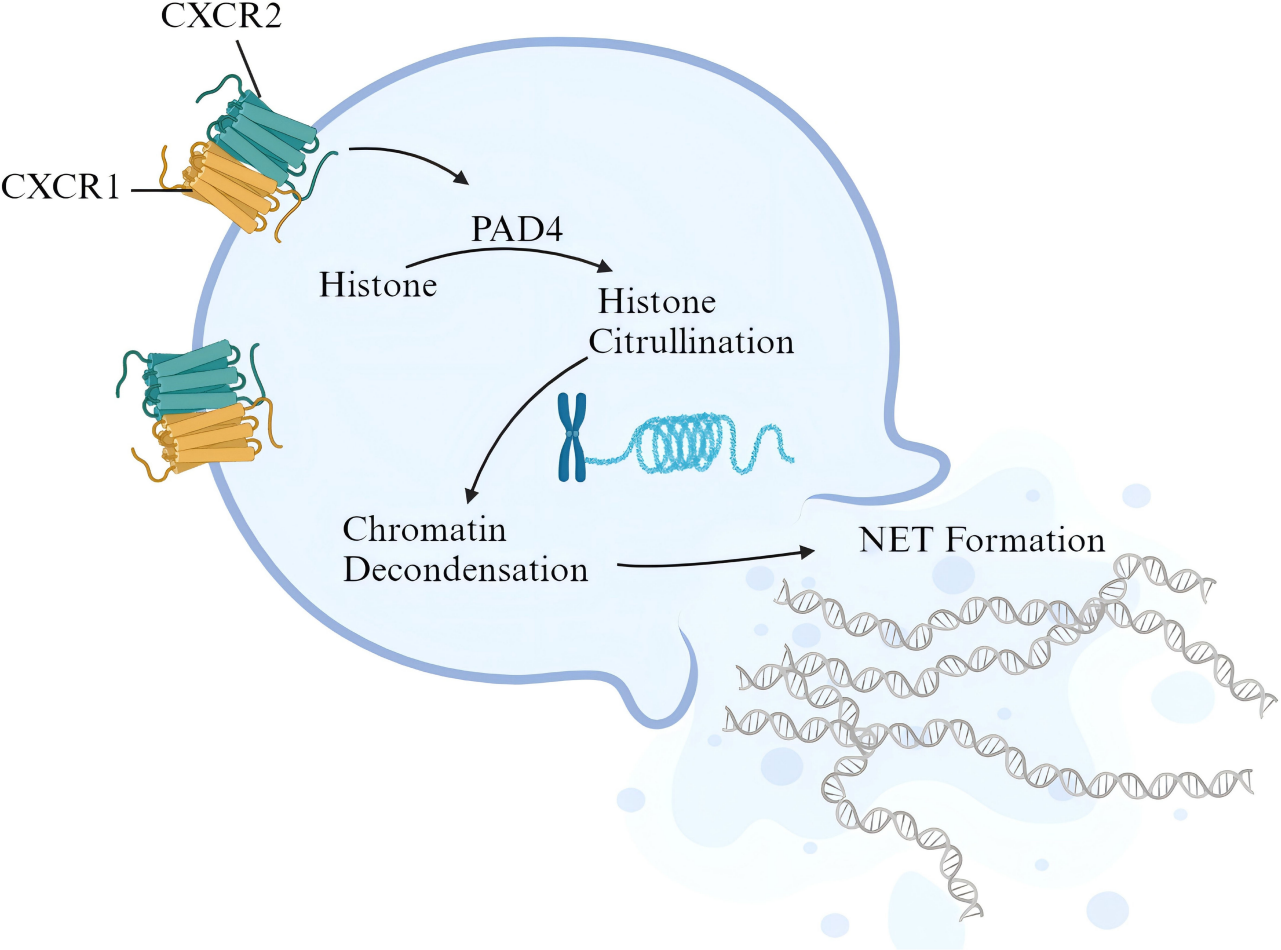
Possible pathogenic mechanism of CXCR1/2 in UC. The figure was created with biorender.com.

At present, there is no valid evidence of the relationship between CXCR1/2 signaling and NET release in active colitis. Nevertheless, it has been observed that activation of CXCR1/2 signaling contributes to NET formation in sepsis patients and animal models. Activated CXCR1/2 signaling can consistently induce NET formation of isolated neutrophils from normal mice. Targeted blocking of CXCR1/2 signaling with reparixin inhibited neutrophil infiltration and NET formation in lung tissue, manifested as the decrease in the content of citrullinated histone H3 (38). PAD4 promotes histone citrullination and is a key enzyme in NET formation. Higher expression of PAD4 generally means the activation of NET formation (23). In expression correlation analysis, we found a positive correlation between CXCR1/2 and PAD4 expression in colonic tissues of UC patients, indicating that CXCR1/2 overexpression in colon tissue may activate PAD4 pathway, then inducing the citrullination of histone to promote NET formation. In conclusion, based on our findings, we identified the pathogenic role of CXCR1 and CXCR2 in UC patients and brought up a possible mechanism. Under the stimulation of chemokines like CXCL8 from inflamed intestine, peripheral neutrophils expressing CXCR1 and CXCR2 are recruited to intestinal tract, and activated to release NETs, thereby promoting downstream pathways of epithelium damage and amplifying inflammatory response (**Figure 10**).

This article also had some limitations. First, although Mendelian randomization excludes the influence of confounding factors, there may be some undiscovered associations between the included SNPs and certain phenotypes, which may lead to potential pleiotropy. In addition, we must acknowledge that there are still some discrepancies between the two cohorts used. In Finngen, mainly hospitalized patients from Finland are included, while in de Lange’s study, GWAS from multiple cohorts from different countries are combined through meta-analysis. Differences in ethnicity and degree of disease in the included populations can certainly lead to discrepant MR results.

## 5 Conclusion

We identified the causal relationships between seven NRGs and UC in this study. The causal effects of CXCR1 and CXCR2 were further supported by the evidence from colocalization. Transcriptomic analysis indicated that CXCR1/2 may play a crucial role in NET-mediated inflammation of UC. Integrating multiple analysis method, our study suggested that strategies targeting the CXCR1/CXCR2 pathway to inhibit NET formation would have promising translational potential in UC.

## Data Availability

The original contributions presented in the study are included in the article/**Supplementary Material**. Further inquiries can be directed to the corresponding author.

## Supplementary material

Supplementary FIGURE S1 The results of leave-one-analysis in exploration phase.

Supplementary FIGURE S2 The results of leave-one-analysis in validation phase.
