# Supplementary figures and images for: CXCR1 and CXCR2 are potential neutrophil extracellular trap-related treatment targets in ulcerative colitis: insights from Mendelian randomization, colocalization and transcriptomic analysis
Source: Front Immunol. 2024 Sep 12;15:1425363. doi: 10.3389/fimmu.2024.1425363 (PMC11424450; doi:10.3389/fimmu.2024.1425363)

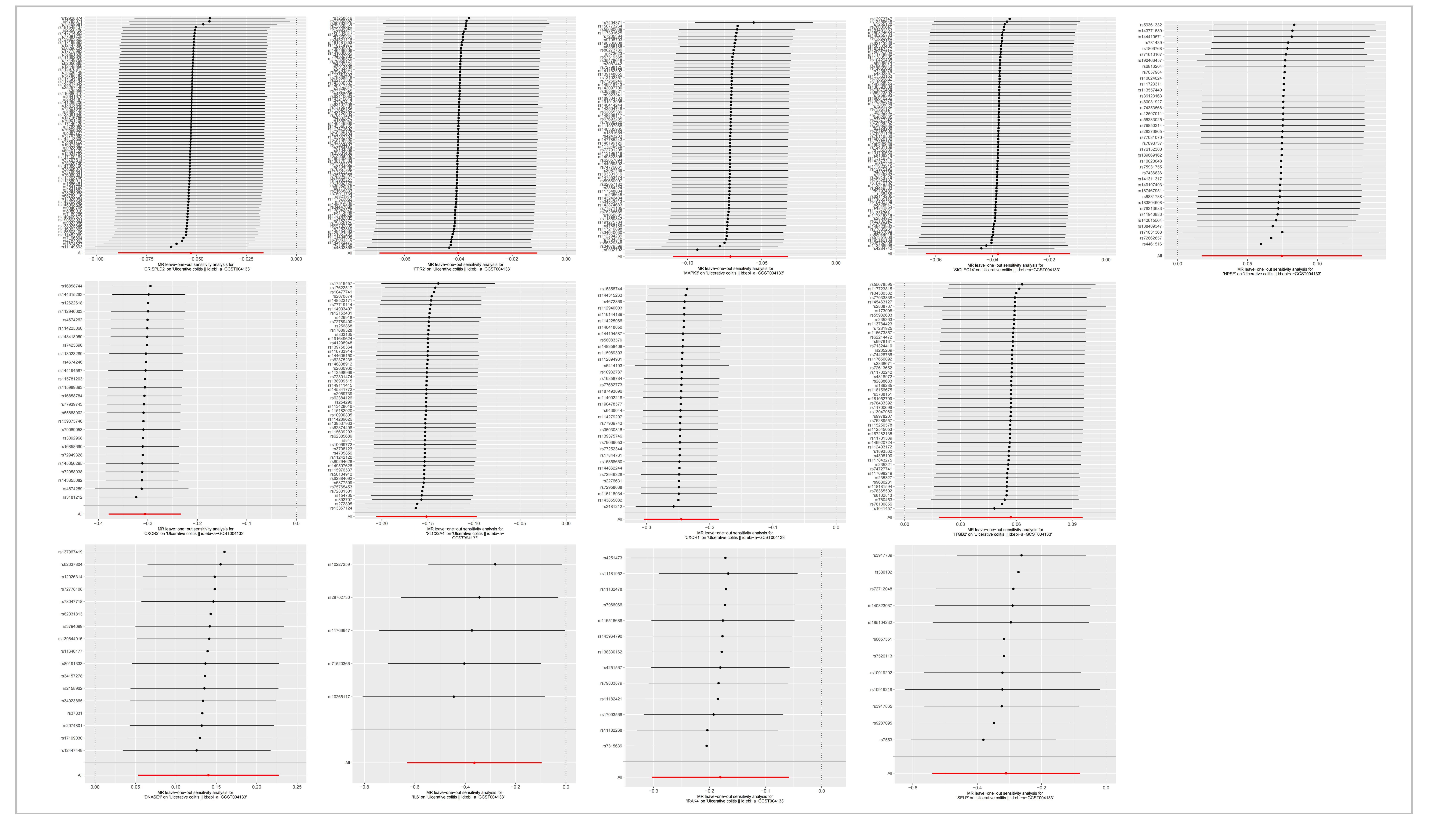

Supplement: Supplementary FIGURE S1 — The results of leave-one-analysis in exploration phase. [file Image1.jpeg]

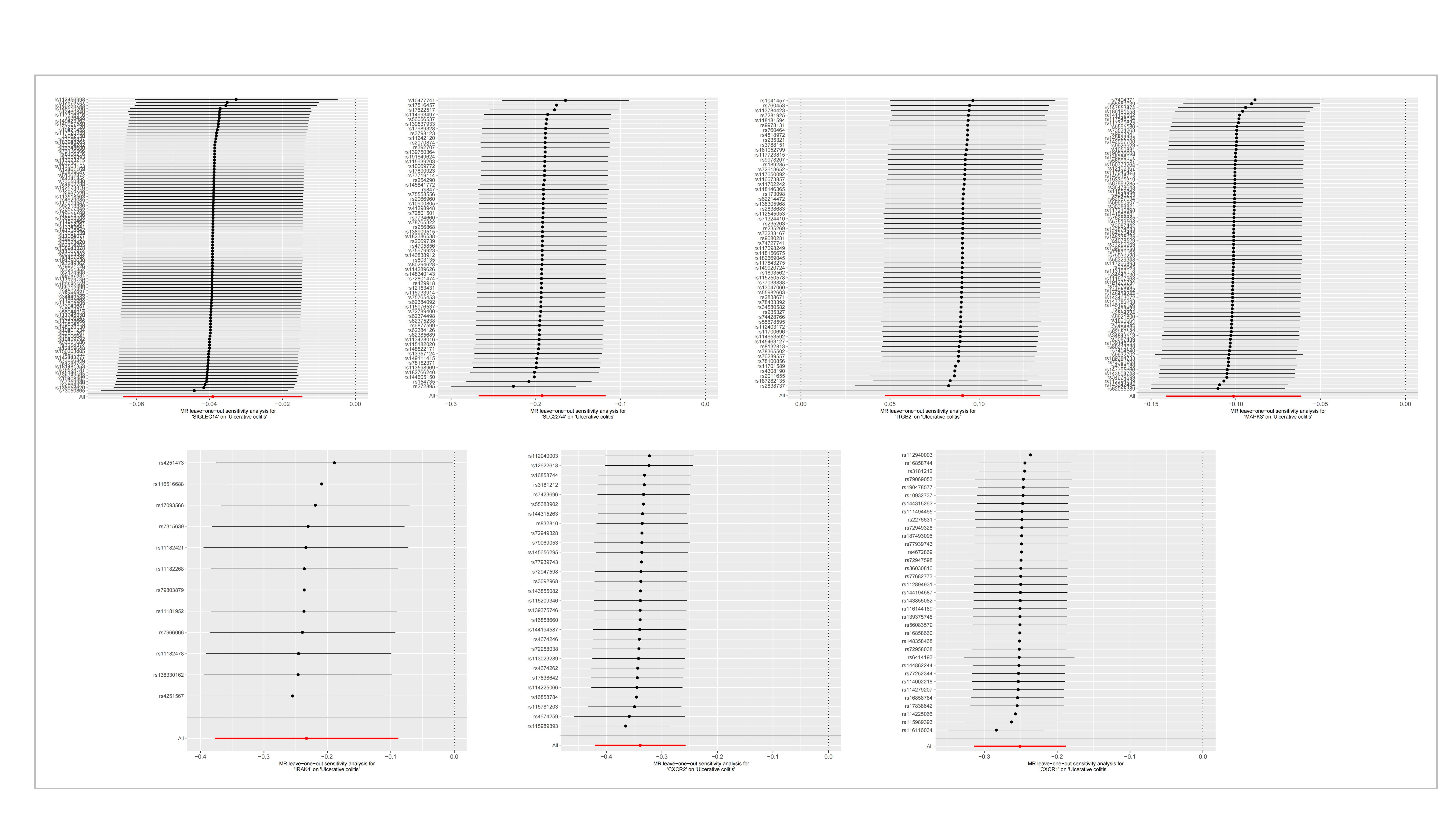

Supplement: Supplementary FIGURE S2 — The results of leave-one-analysis in validation phase. [file Image2.jpeg]
